# Supplementary material for: Altered glycolysis triggers impaired mitochondrial metabolism and mTORC1 activation in diabetic β-cells
Source: Nat Commun. 2022 Nov 14;13:6754. doi: 10.1038/s41467-022-34095-x (PMC9663558; doi:10.1038/s41467-022-34095-x)

Supplementary Information for

Altered glycolysis triggers impaired mitochondrial metabolism and mTORC1 activation in diabetic β-cells

by

Elizabeth Haythorne, Matthew Lloyd, John Walsby-Tickle, Andrei Tarasov, Jonas Sandbrink, Idoia Portillo, Raul Terron Exposito, Gregor Sachse, Malgorzata Cyranka, Maria Rohm, Patrik Rorsman, James McCullagh and Frances M Ashcroft

Supplementary Figures 1-7
Table 1A
Table 1B
Uncropped blots for Supplementary Figures 4 and 5

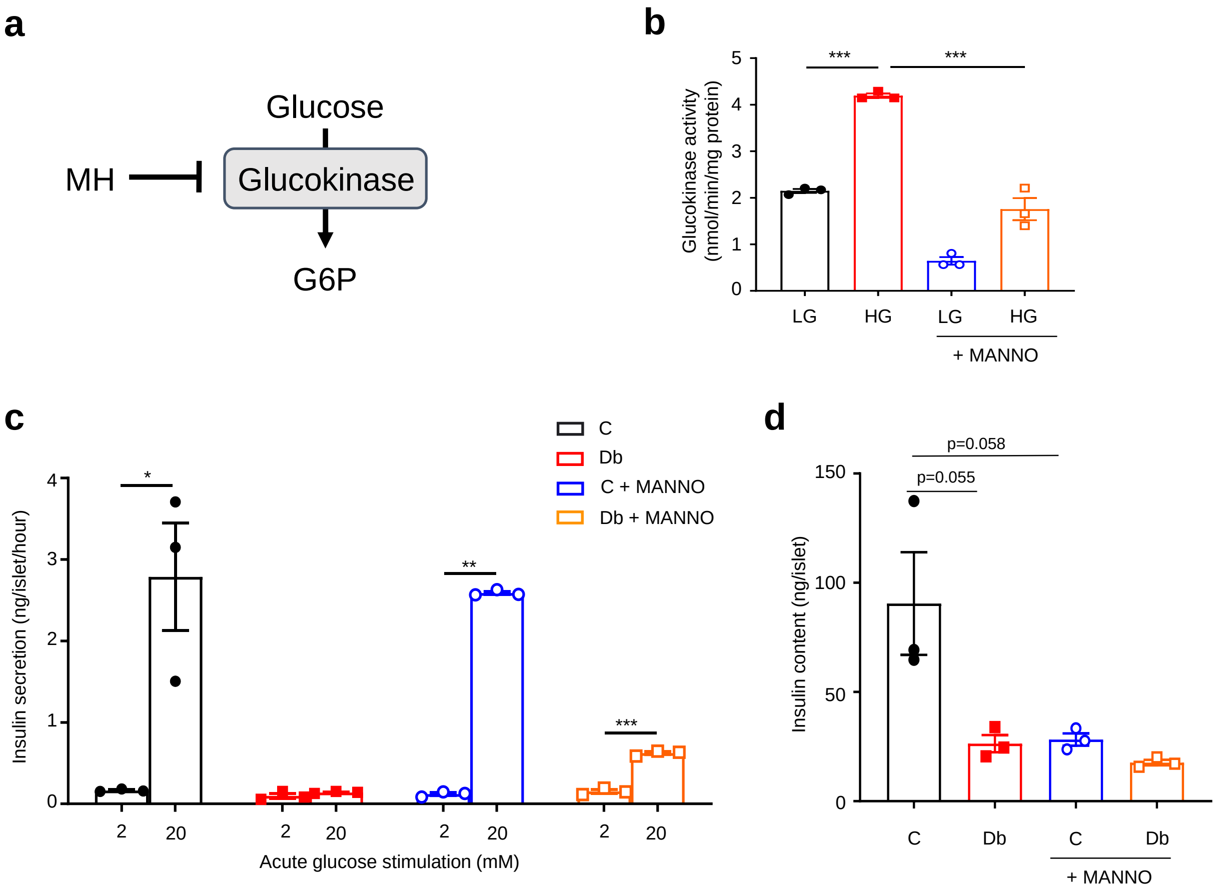

Supplementary Figure 1. Mannoheptulose partially restores insulin secretion in diabetic islets

(**a**) Diagram showing where mannoheptulose (MH) inhibits glucose metabolism.

(**b**) Summed activity of all hexokinases measured in LG- or HG-cells incubated for 48hr ± 10mM mannoheptulose. Activity was measured at 20mM glucose, where glucokinase activity predominates, in the continued presence of 10mM mannoheptulose (MANNO) (n=3 biologically independent experiments).

(**c,d**). Insulin secretion (**c**) and insulin content (**d**) in control (C) and diabetic (Db) islets incubated for 48hr ± 10mM mannoheptulose and then measured at 2mM or 20mM glucose for 1h in the absence of mannoheptulose (n=3 animals/genotype).

All panels show individual data points and mean ± s.e.m. *p < 0.05, **p<0.01, ***p < 0.001, two-tailed unpaired Student's t-test. Control (black), diabetic (red), control + MANNO (blue), diabetic + MANNO (orange). Source data are provided in the Supplementary Source Data file.

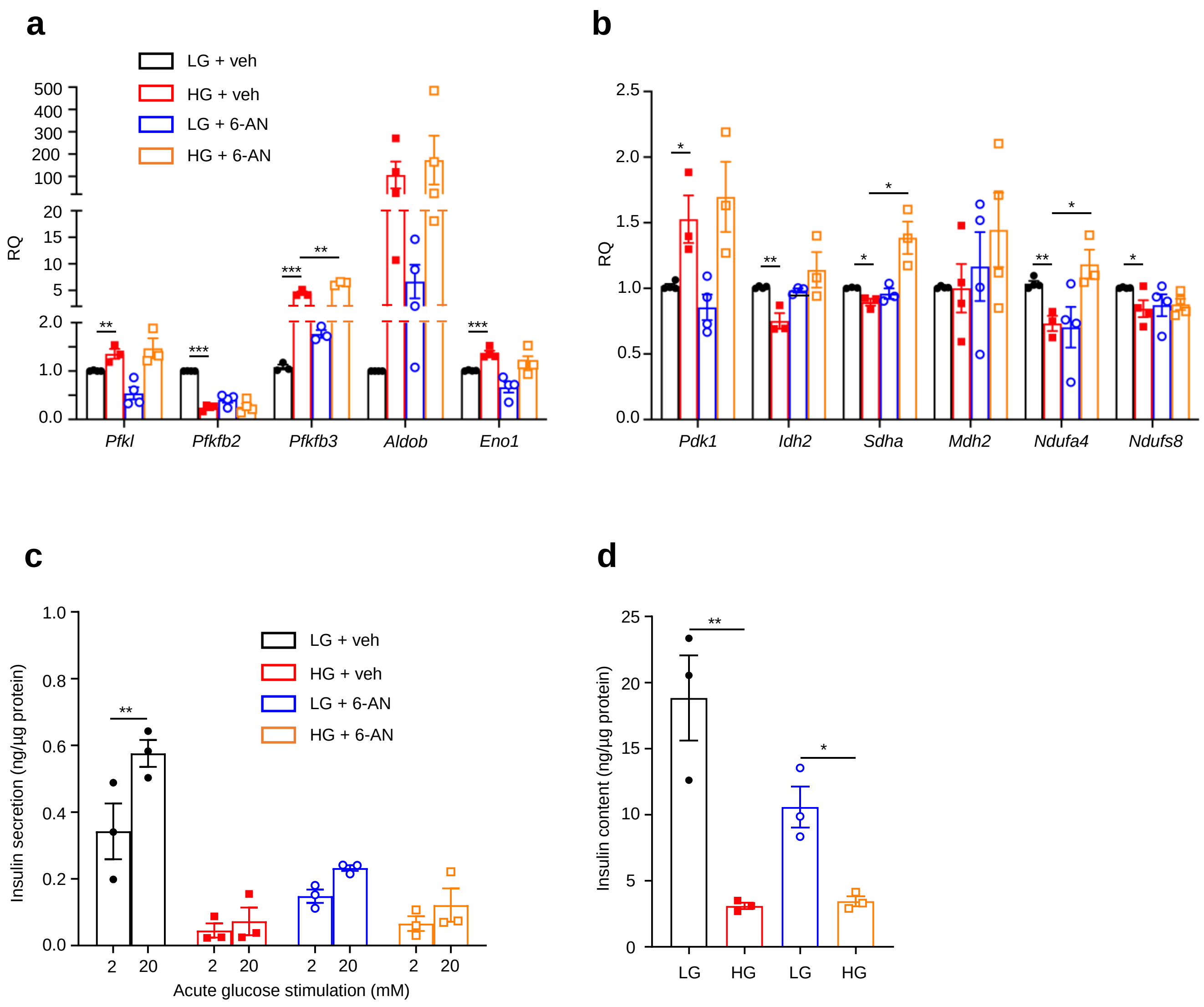

Supplementary Figure 2. Inhibition of the pentose phosphate pathway does not prevent gene changes and loss of insulin secretion in HG-cells.

(a,b) mRNA levels of the indicated genes involved in glycolytic (**a**) and mitochondrial (**b**) metabolism assessed by qPCR in LG-cells and HG-cells cultured for 48hrs + 0.1% DMSO (veh) or 100µM 6-aminonicotinamide (6-AN). *Pfkfb2, AldoB, Eno1, Mdh2, Ndufs8,* n=4 independent experiments; *Pfkfb3, Idh2, Sdha,* n=3 biologically independent experiments; *Pfkl, Pdk1, Ndufa4*, n=4 biologically independent experiments for LG and LG+6-AN, but n=3 biologically independent experiments for HG and HG+6-AN.

(**c,d**) Insulin secretion (**c**) and insulin content (**d**) in LG-cells and HG-cells cultured for 48hrs + 0.1% DMSO (veh) or 100µM 6-aminonicotinamide (6-AN) and then exposed to 2mM or 20mM glucose for 1 hr (n=3 biologically independent experiments).

All panels show individual data points and mean ± s.e.m. *p < 0.05, **p<0.01, ***p < 0.001, two-tailed unpaired Student's t-test. Source data are provided in the Supplementary Source Data file.

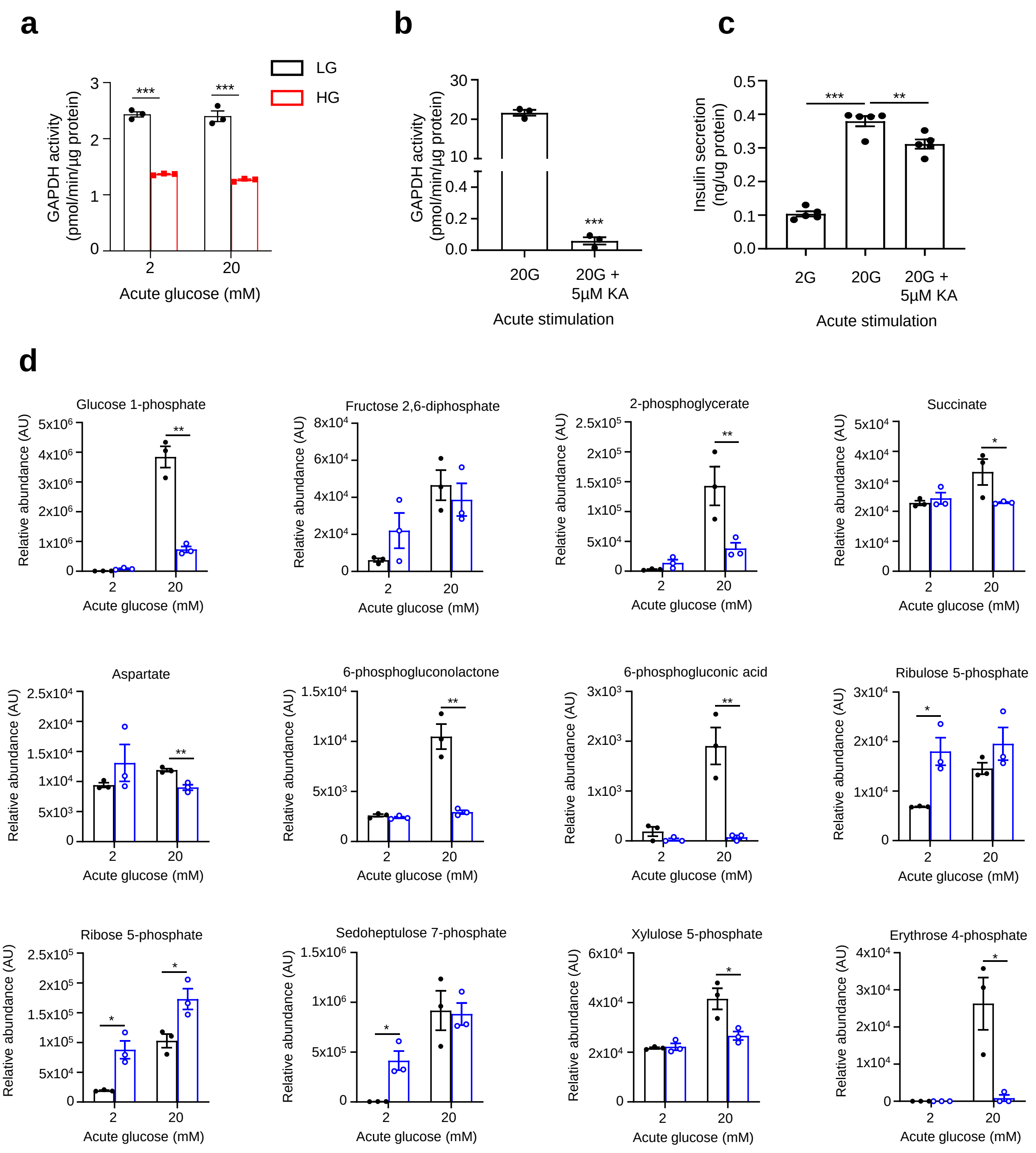

Supplementary Figure 3. Inhibition of GAPDH induces gene changes to similar those induced by chronic hyperglycaemia

(**a**) GAPDH activity in LG-cells (black) and HG-cells (red) (n=3 biologically independent experiments).

(**b,c**) Acute effects of 5µM koningic acid (KA) on GAPDH activity (**b**, n=3 independent experiments) and insulin secretion (**c,** n=5 biologically independent experiments). LG-cells were exposed to KA for 30mins.

(d) Metabolite abundances in LG-cells cultured for 48 hr in the absence (black) or presence (blue) of 5µM koningic acid (KA) and subsequently stimulated with 2mM or 20mM glucose for 1h in the absence of KA (n=3 biologically independent experiments).

All panels show individual data points and mean ± s.e.m. *$p < 0.05$, **$p<0.01$, ***$p < 0.001$, two-tailed unpaired Student's t-test. Source data are provided in the Supplementary Source Data file.

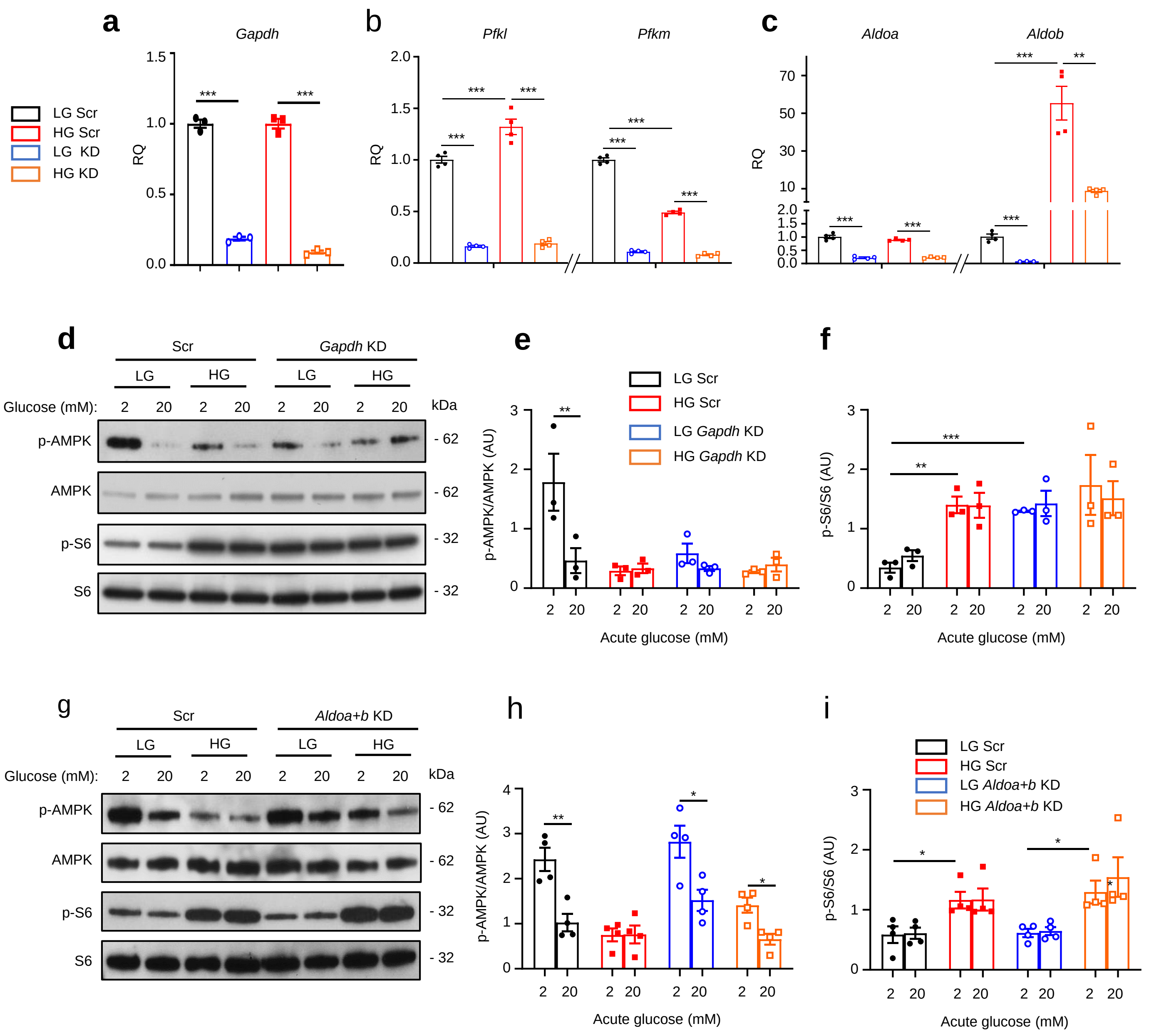

Supplementary Figure 4. Effects of GAPDH and Aldolase knockdown on AMPK and mTOR

(**a**) *Gapdh* mRNA level in LG-cells and HG-cells cultured for 48hrs with either scrambled siRNA (Scr) or *Gapdh* siRNA (KD) (n=3 biologically independent experiments).

(**b**) *Pfkl* and *Pfkm* mRNA levels in LG-cells and HG-cells cultured for 48hrs with either scrambled siRNA (Scr) or both *Pfkl* and *Pfkm* siRNAs (KD) (n=4 biologically independent experiments).

(**c**) *Aldoa* and *Aldob* mRNA levels in LG-cells and HG-cells cultured for 48hrs with either scrambled siRNA (Scr) or both *Aldoa* and *Aldob* siRNAs (KD) (n=4 biologically independent experiments).

(**d**) Representative Western blot of lysates from cells transfected with scrambled siRNA (Scr) or *Gapdh* siRNA (*Gapdh* KD) and cultured at low (LG) or high (HG) glucose for 48h. Cells were subsequently stimulated with 2mM or 20mM glucose for 1h. Phosphorylated (p) and total AMPK and S6. (**e**, **f**) Quantitative densitometry analysis of p-AMPK/AMPK (**e**, n=3 independent experiments) and p-S6/S6 (**f**, n=3 biologically independent experiments).

(**g**) Representative Western blot of lysates from cells transfected with scrambled siRNA (Scr) or *Aldoa* and *Aldob* siRNA (*Aldoa+b* KD) and cultured at low (LG) or high (HG) glucose. Cells were subsequently stimulated with 2mM or 20mM glucose for 1h. Phosphorylated (p) and total AMPK and S6. (**h,i**) Quantitative densitometry analysis of p-AMPK/AMPK (**h**, n=4 biologically independent experiments) and p-S6/S6 (**i**, n=4 biologically independent experiments).

All panels show individual data points and mean ± s.e.m. *p < 0.05, **p<0.01, ***p < 0.001, two-tailed unpaired Student's t-test. Uncropped Western blots are provided at the end of this file. Source data are provided in the Supplementary Source Data file.

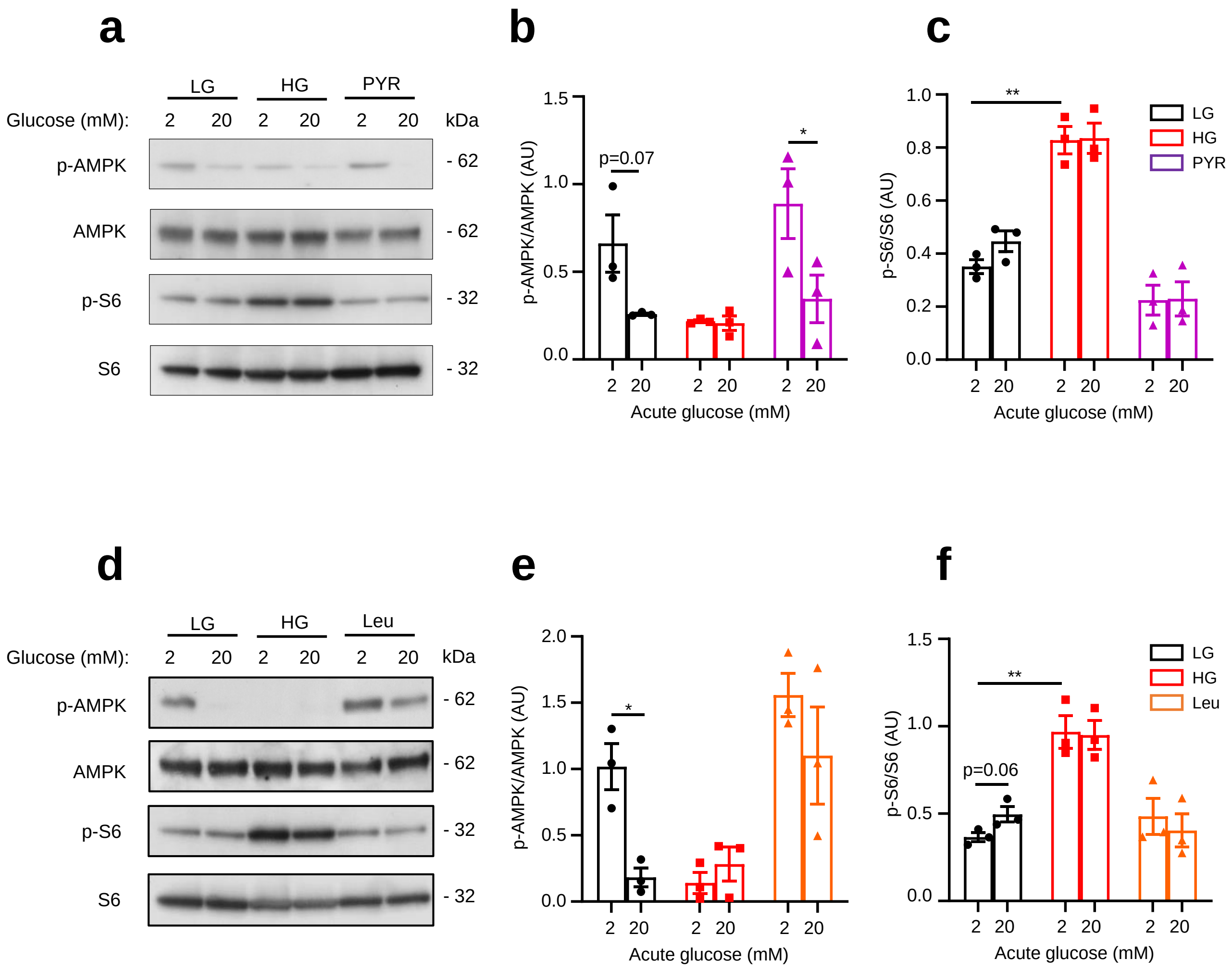

Supplementary Figure 5. Effects of chronic Me-pyruvate and leucine on AMPK and mTOR

(**a**) Representative Western blot of lysates from LG-cells, HG-cells and cells cultured for 48hr in 20mM Me-pyruvate (PYR), and then stimulated with 2mM or 20mM glucose for 1hr. Phosphorylated (p) and total AMPK and S6.

(**b,c**) Quantitative densitometry analysis of p-AMPK/AMPK (**b**, n=3 biologically independent experiments) and p-S6/S6 (**c**, n=3 biologically independent experiments). LG-cells (black), HG-cells (red), 20mM Me-pyruvate (purple).

(**d**) Representative Western blot of lysates from LG-cells, HG-cells and cells cultured for 48hr in 20mM leucine (Leu), then stimulated with 2mM or 20mM glucose for 1hr. Phosphorylated (p) and total AMPK and S6.

(**e,f**) Quantitative densitometry analysis of p-AMPK/AMPK (**e**, n=3 biologically independent experiments) and p-S6/S6 (**f**, n=3 biologically independent experiments). LG-cells (black), HG-cells (red), 20mM leucine (orange).

Data are individual data points plus mean±sem. *$p < 0.05$, **$p<0.01$, two-tailed unpaired Student's t-test. Uncropped Western blots are provided at the end of this file. Source data are provided in the Supplementary Source Data file.

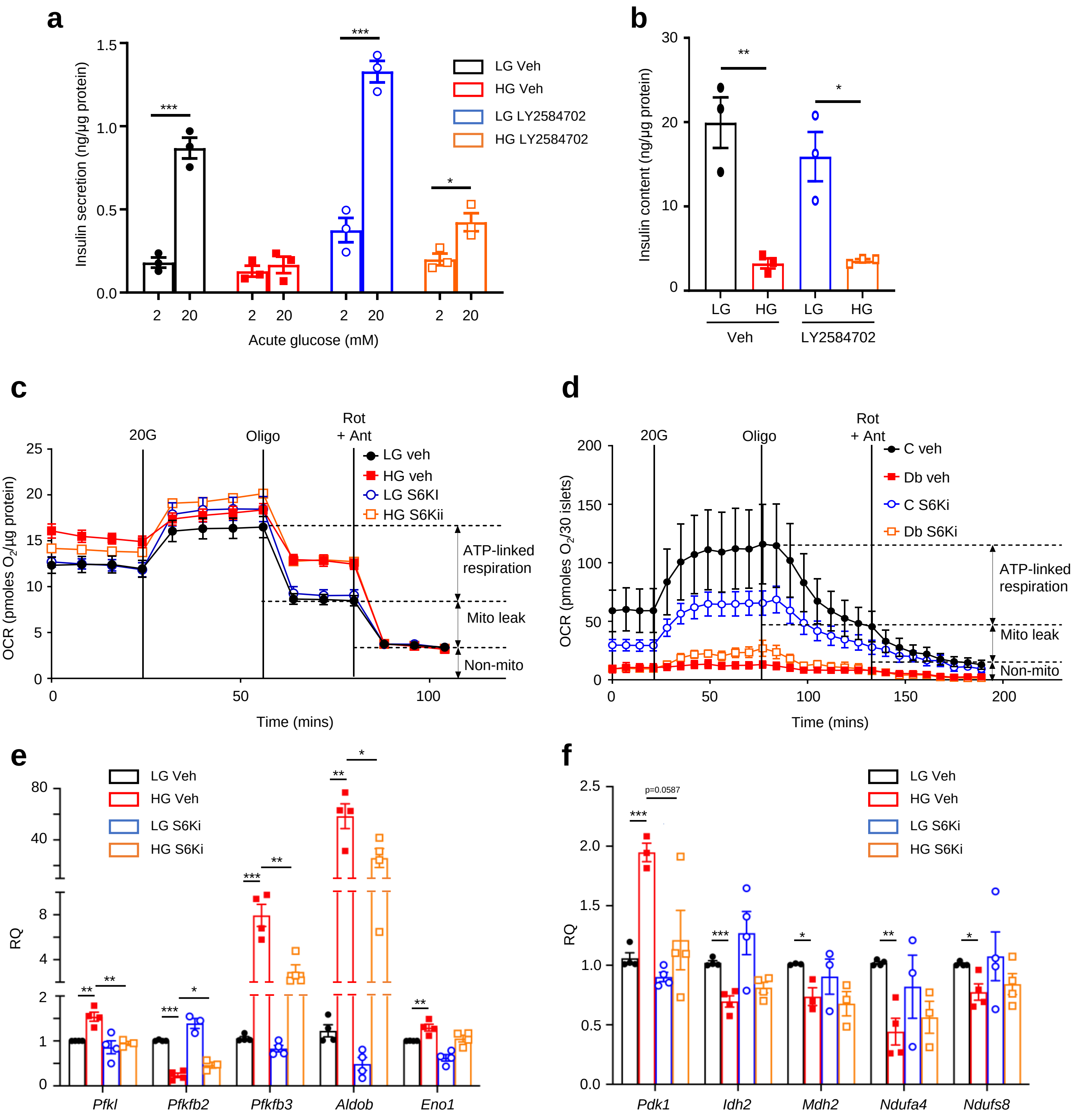

Supplementary Figure 6. Inhibition of S6 kinase partially prevents the effects of chronic hyperglycaemia

For legend see next page.

Supplementary Figure 6. Inhibition of S6 kinase partially prevents the effects of chronic hyperglycaemia

(**a,b**) Insulin secretion (**a**) and insulin content (**b**) in LG-cells and HG-cells cultured for 48hr in the presence of absence of 10µM of the S6 kinase inhibitor, LY2584702 (n=3 independent experiments). (**b**) Oxygen consumption rate (OCR) of LG- and HG-cells cultured for 48hr ± 10µM of the S6 kinase inhibitor PF-4708671 (S6Ki). OCR is shown at 2mM glucose and after sequential addition of 20mM glucose (20G), 1 µM oligomycin (Oligo) and 0.5µM rotenone + 0.5µM antimycin A (Rot+Ant) (n=5 biologically independent experiments/group).

(**c**) Oxygen consumption rate (OCR) of control and diabetic islets cultured for 48hr ± 10µM S6Ki. OCR is shown at 2 mM glucose and after sequential addition of 20mM glucose (20G), 5 µM oligomycin (Oligo) and 5 µM rotenone + 5 µM antimycin A (Rot+Ant). Control islets (black, n=7 mice examined over 7 independent experiments), Diabetic islets (red, n=7 animals examined over 7 independent experiments), Control islets + S6Ki (blue, n=7 mice examined over 10 independent experiments), Diabetic islets + S6Ki (orange, n=7 mice examined over 8 independent experiments).

(e,f) mRNA levels for the indicated genes involved in glycolytic (**e**) and mitochondrial (**f**) metabolism assessed by qPCR in LG-cells and HG-cells cultured for 48hrs ± 10µM of the S6-kinase inhibitor PF-4708671 (S6Ki). *Pfkfb3, Aldob, Eno1, Idh2,* n=4 biologically independent experiments for all conditions; *Mdh2*, n=3 biologically independent experiments for all conditions; *Pfkfb2*, *Ndufa4*, n=4 biologically independent experiments for LG+Veh and HG+Veh but n=3 biologically independent experiments for LG+S6Ki and HG+S6Ki; *Pfkl*, n=4 biologically independent experiments for LG+Veh, HG+Veh and LG+S6KI but n=3 biologically independent experiments for HG+S6Ki. *Pdk1*, n=4 biologically independent experiments for LG+Veh, LG+S6Ki and HG+S6KI but n=3 biologically independent experiments for HG+Veh

All panels show mean ± s.e.m. *p < 0.05, **p<0.01, ***p < 0.001; two-tailed unpaired Student's t-test. Source data are provided in the Supplementary Source Data file.

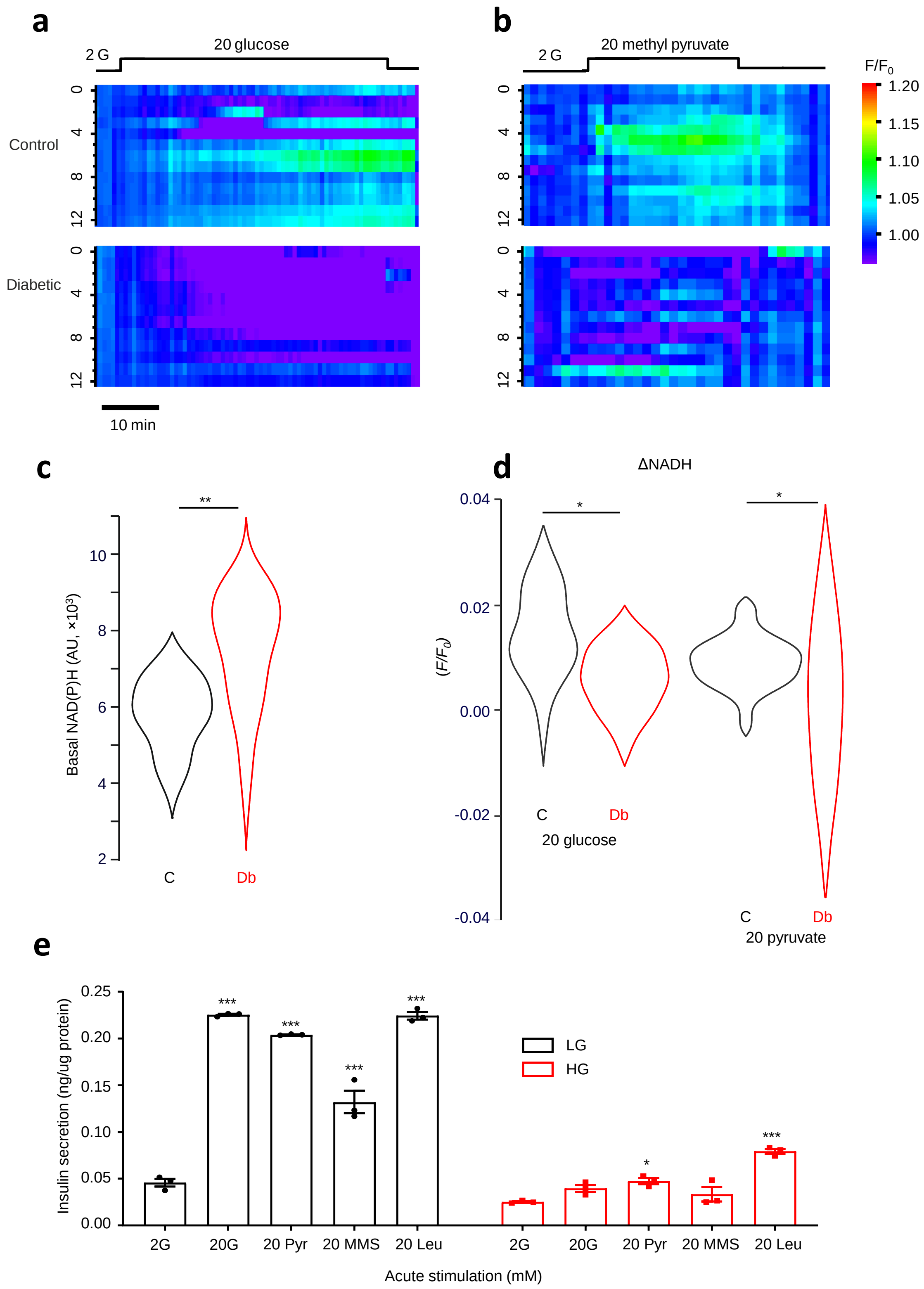

Supplementary Figure 7
For legend see next page

Supplementary Figure 7. Diabetic islets display an attenuated response to glycolytic and mitochondrial substrates

(**a,b**) Representative heatmaps of the time course of NADH autofluorescence in islets isolated from control and βV59M diabetic mice, as indicated, in response to (**a**) a glycolytic (20 mM glucose, 20G) or (**b**) a mitochondrial (20 mM methyl-pyruvate) substrate.

(**c,d**) Quantification of the magnitude of NADH autofluorescence for control (C) and diabetic (Db) islets. (**c**) Basal (2 mM glucose) autofluorescence of NAD(P)H in arbitrary units. n= 71 control islets and n=140 diabetic islets. As control and diabetic islets were imaged in the same field arbitrary units can be compared. (**d**) Fold change in NAD(P)H fluorescence intensity induced by 20 mM glucose (n=83 control islets and n=150 diabetic islets) or 20 mM methyl pyruvate (n=54 control and n=78 diabetic islets).

(e) Insulin secretion in LG-cells and HG-cells stimulated for 1h with 2mM glucose or 20mM glucose (G), 20mM methyl pyruvate (MeP), 20mM monomethyl succinate (MMS) or20mM leucine (leu) (n=3 biologically independent experiments).

All panels show mean ± s.e.m. *p < 0.05, **p<0.01; (c-d), two-tailed unpaired Student's t-test. (e), two-way ANOVA with Bonferroni post hoc corrections. Source data are provided at the end of this file.

Antibody	Source	Dilution
Rabbit anti-phospho-AMPKα-T172 antibody	Cell Signaling Technology, cat. #2535	1:1000
Rabbit anti-AMPKα antibody	Cell Signaling Technology, cat. #2532	1:1000
Rabbit anti-phospho-S6-S240/244 antibody	Cell Signaling Technology, cat. #5364	1:4000
Rabbit anti-S6 antibody	Cell Signaling Technology, cat. #2217	1:4000
Rabbit anti-phospho-PDHe1α-S293	Cell Signaling Technology, cat. #31866	1:1000
Rabbit anti-αtubulin antibody	Cell Signaling Technology, cat. #2144S	1:1000
Rabbit anti-phospho-Raptor (Ser792) Antibody	Cell Signaling Technology, cat. #2083	1:1000
Rabbit anti-Raptor Antibody	Cell Signaling Technology, cat. #2280	1:1000
Rabbit anti-phospho-4E-BP1 (Thr37/46) Antibody	Cell Signaling Technology, cat. #2855	1:1000
Rabbit anti-4E-BP1 Antibody	Cell Signaling Technology, cat. #9644	1:1000

Supplementary Table 1 A. Western blot antibodies

Gene	Catalogue number (Applied Biosystems)
Actb	Rn00667869_m1
Aldoa	Rn00820577_g1
Aldob	Rn01768292_m1
Eno1	Rn01518942_gH
Gapdh	Rn01775763_g1
Hprt1	Rn01527840_m1
Hspa8	Rn00821191_g1
Idh2	Rn01478119_m1
Mdh2	Mm00725890_s1
Ndufa4	Mm00809672_s1
Ndufs8	Mm00523063_m1
Pdk1	Mm00554306_m1
Pfkfb2	Mm00435575_m1
Pfkfb3	Rn00678825_m1
Pfkl	Mm00435587_m1
Pfkm	Mm01309576_m1
Sdha	Rn00590475_m1

Supplementary Table 1B. Taqman probes

Uncropped western blots for Supplementary Fig.4d

phospho-AMPK (red box refers to bands in Fig.S4d)

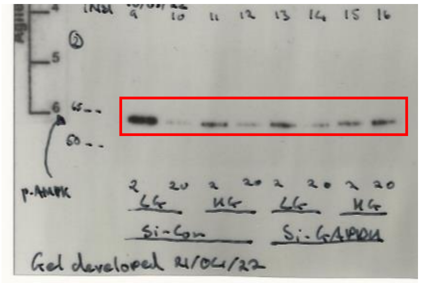

AMPK (red box refers to bands in Fig.S4d)

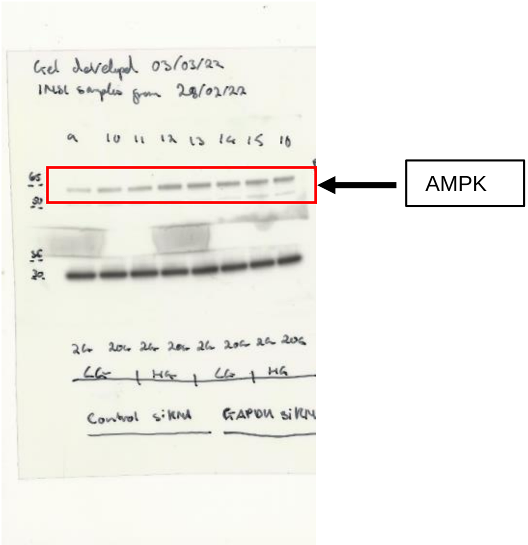

AMPK

phospho-S6 (red box refers to bands in Fig.S4d, bands at 62 kDa are from different antibody)

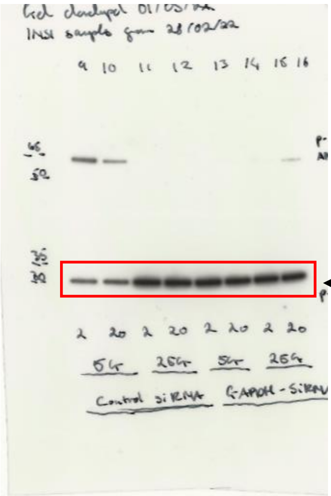

phospho-S6

S6 (red box refers to bands in Fig.S4d)

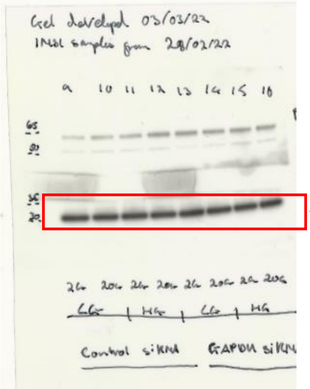

S6

Uncropped western blots for Supplementary Fig.4g

phospho-AMPK (red box refers to bands in Fig.S4g)

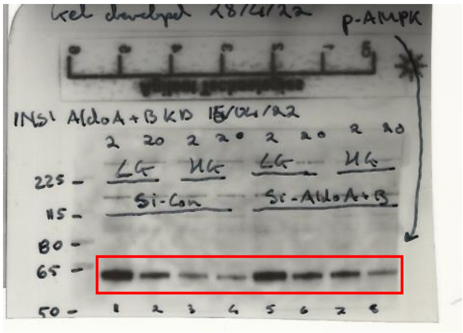

AMPK (red box refers to bands in Fig.S4g)

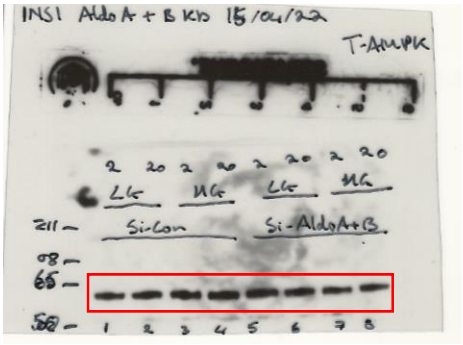

phospho-S6 (red box refers to bands in Fig.S4g)

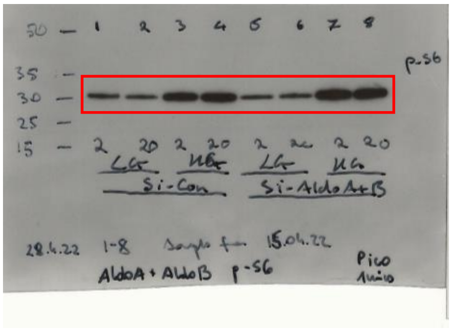

S6 (red box refers to bands in Fig.S4g)

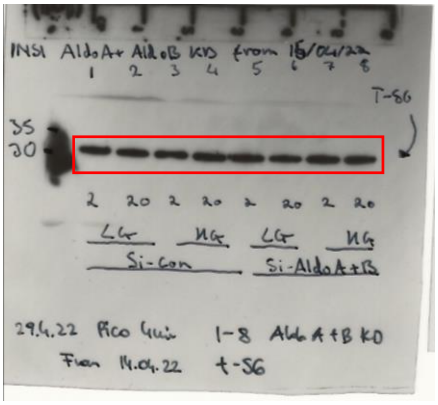

Uncropped western blots for Supplementary Fig.5a

phospho-AMPK (red box refers to bands in Fig.S5a, bands at 32 kDa are from separate antibody on separate membrane)

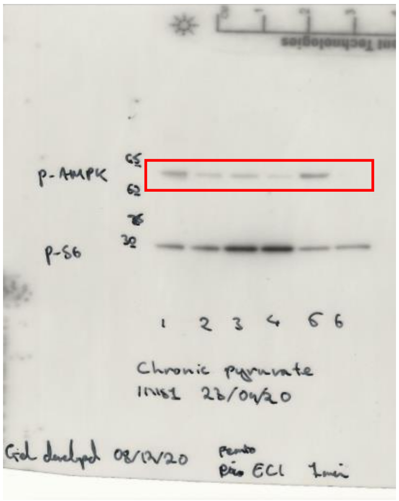

AMPK (red box refers to bands in Fig.S5a)

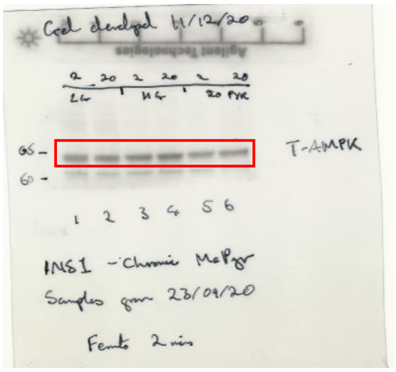

phospho-S6 (red box refers to bands in Fig.S5a, bands at 62 kDa are from separate antibody on separate membrane)

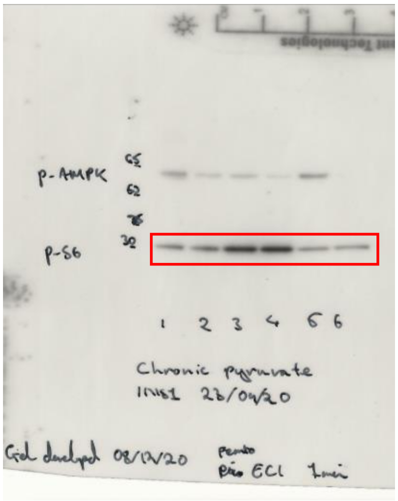

S6 (red box refers to bands in Fig.S5a)

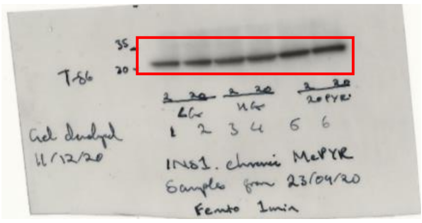

Uncropped western blots for Supplementary Fig.5d

phospho-AMPK (red box refers to bands in Fig.S5d)

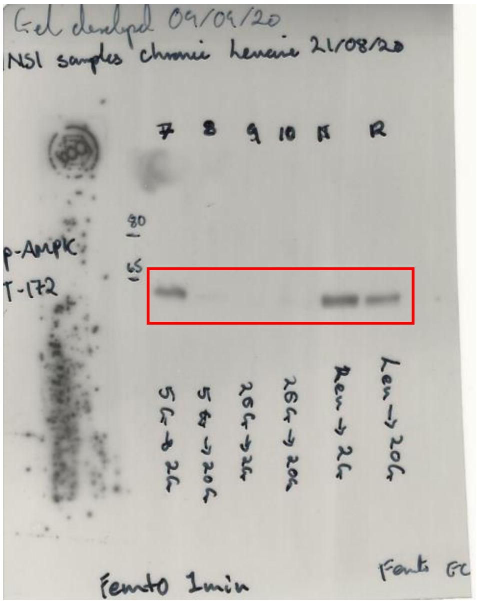

AMPK (red box refers to bands in Fig.S5d)

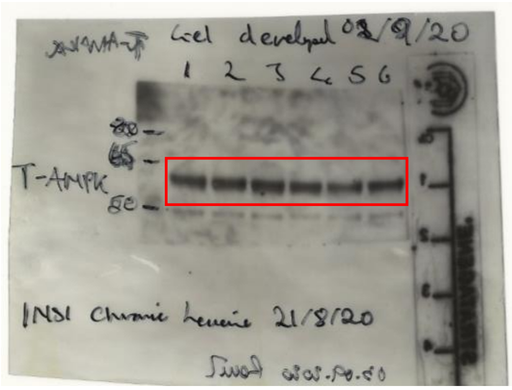

phospho-S6 (red box refers to bands in Fig.S5d)

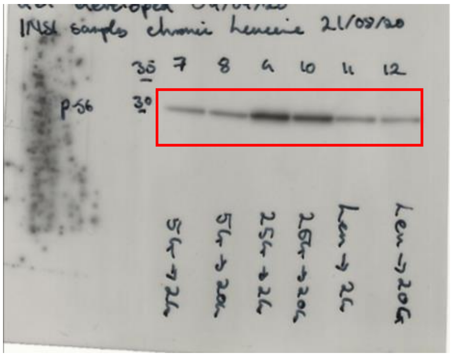

S6 (red box refers to bands in Fig.S5d, bands at 62 kDa are from separate antibody on separate membrane)

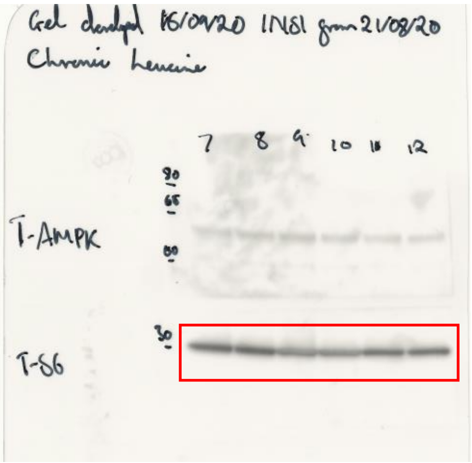

Supplement: Supplementary file 1 — Supplementary Information [file 41467_2022_34095_MOESM1_ESM.pdf]
