# Supplementary material for: Altered glycolysis triggers impaired mitochondrial metabolism and mTORC1 activation in diabetic β-cells
Source: Nat Commun. 2022 Nov 14;13:6754. doi: 10.1038/s41467-022-34095-x (PMC9663558; doi:10.1038/s41467-022-34095-x)
Supplement: Supplementary file 3 — Description of Additional Supplementary Files [file 41467_2022_34095_MOESM3_ESM.pdf]

File name: Supplementary Data 1A-C and 2A-B

Description: Metabolomics data sets associated with Figure 3.

Supplementary Data File 1A: relative abundance of metabolites at 2mM and 20mM glucose in wild-type islets

Supplementary Data File 1B: relative abundance of metabolites at 2mM glucose in wild-type and diabetic islets

Supplementary Data File 1C: relative abundance of metabolites at 20mM glucose in wild-type and diabetic islets

Supplementary Data File 2A: relative abundance of metabolites at 2mM glucose in high glucose cultured INS1 cells (HG cells) and low glucose cultured INS 1 cells (LG cells)

Supplementary Data File 2B: relative abundance of metabolites at 20mM glucose in high glucose cultured INS1 cells (HG cells) and low glucose cultured INS 1 cells (LG cells)
