# Supplementary material for: Altered glycolysis triggers impaired mitochondrial metabolism and mTORC1 activation in diabetic β-cells
Source: Nat Commun. 2022 Nov 14;13:6754. doi: 10.1038/s41467-022-34095-x (PMC9663558; doi:10.1038/s41467-022-34095-x)
Supplement: Supplementary file 5 — Reporting Summary [file 41467_2022_34095_MOESM5_ESM.pdf]

nature portfolio

Corresponding author(s): Frances Ashcroft

Last updated by author(s): 21/09/22

Reporting Summary

Nature Portfolio wishes to improve the reproducibility of the work that we publish. This form provides structure for consistency and transparency in reporting. For further information on Nature Portfolio policies, see our Editorial Policies and the Editorial Policy Checklist.

Statistics

For all statistical analyses, confirm that the following items are present in the figure legend, table legend, main text, or Methods section.

n/a	Confirmed	
☐	☒	The exact sample size (*n*) for each experimental group/condition, given as a discrete number and unit of measurement
☐	☒	A statement on whether measurements were taken from distinct samples or whether the same sample was measured repeatedly
☐	☒	The statistical test(s) used AND whether they are one- or two-sided<br>*Only common tests should be described solely by name; describe more complex techniques in the Methods section.*
☒	☐	A description of all covariates tested
☐	☒	A description of any assumptions or corrections, such as tests of normality and adjustment for multiple comparisons
☐	☒	A full description of the statistical parameters including central tendency (e.g. means) or other basic estimates (e.g. regression coefficient) AND variation (e.g. standard deviation) or associated estimates of uncertainty (e.g. confidence intervals)
☐	☒	For null hypothesis testing, the test statistic (e.g. *F*, *t*, *r*) with confidence intervals, effect sizes, degrees of freedom and *P* value noted<br>*Give P values as exact values whenever suitable.*
☒	☐	For Bayesian analysis, information on the choice of priors and Markov chain Monte Carlo settings
☒	☐	For hierarchical and complex designs, identification of the appropriate level for tests and full reporting of outcomes
☒	☐	Estimates of effect sizes (e.g. Cohen's *d*, Pearson's *r*), indicating how they were calculated

Our web collection on statistics for biologists contains articles on many of the points above.

Software and code

Policy information about availability of computer code

Data collection	Metabolomics: Thermo Xcalibur 4.2<br>Fluorescent imaging: Zeiss ZenBlue (2012 version)
Data analysis	Metabolomics: Thermo Xcalibur 4.2, Nonlinear Dynamics Progenesis QI v 2.0, MetaboAnalyst 5.0<br>Fluorescent imaging: Fiji (ImageJ1.53f)<br>Statistics: Graphpad Prism 9.0, Igor Pro 8 (Wavemetrics), R

For manuscripts utilizing custom algorithms or software that are central to the research but not yet described in published literature, software must be made available to editors and reviewers. We strongly encourage code deposition in a community repository (e.g. GitHub). See the Nature Portfolio guidelines for submitting code & software for further information.

Data

Policy information about availability of data

All manuscripts must include a data availability statement. This statement should provide the following information, where applicable:

- Accession codes, unique identifiers, or web links for publicly available datasets
- A description of any restrictions on data availability
- For clinical datasets or third party data, please ensure that the statement adheres to our policy

The raw mass spectrometry data files from which the results were generated for Fig.3a,b, Fig.4a,b, Supplementary Fig.2a,b,c and Supplementary Fig.3a,b have been deposited in Oxford Research Archive, and are available at https://ora.ox.ac.uk/objects/uuid:1859eafb-6fff-4ae6-ba61-f4d224e0ae51 with the DOI: 10.5287/bodleian:dmOmXAVJ5 (resolving to https://doi.org/10.5287/bodleian:dmOmXAVJ5). The authors declare that all data supporting the findings of this study are available within the paper, its Supplementary Information files, its Supplementary Data files or the Source data file.

Field-specific reporting

Please select the one below that is the best fit for your research. If you are not sure, read the appropriate sections before making your selection.

☒ Life sciences ☐ Behavioural & social sciences ☐ Ecological, evolutionary & environmental sciences

For a reference copy of the document with all sections, see nature.com/documents/nr-reporting-summary-flat.pdf

Life sciences study design

All studies must disclose on these points even when the disclosure is negative.

Sample size	The samples sizes for each experiment are stated in the Methods section and the Figure legends of the paper. The sample size for mice was influenced by the requirement to reduce animal usage to the minimal needed to obtain statistical significance, in accordance with UK Home Office guidelines. Power calculations were performed for mouse numbers, based on previous studies (G*power 3.1 software). Sample sizes for experiments on cell lines were based on significance data obtained from similar studies conducted previously.
Data exclusions	No data were excluded
Replication	All experiments were independently performed at least 3 times. In many cases we performed a greater number of independent experiments (eg.4, 6). The number of independent experiments is stated in the individual figure legends and is also detailed in the Source data files. Normally, at least 3 technical replicates were conducted for each experiment. Reproducibility of experimental findings was confirmed by repeating experiments with different experimenters.
Randomization	Randomization was not possible as samples were allocated into experimental groups based on genotype or cell culture conditions (e.g. high or low glucose).
Blinding	For animal/islet experiments the experimenter was blinded to the genotype of the mouse, but not otherwise. All experiments were carried out by at least 2 investigators, therefore blinding for cell experiments was applied at either data collection or data analysis.

Reporting for specific materials, systems and methods

We require information from authors about some types of materials, experimental systems and methods used in many studies. Here, indicate whether each material, system or method listed is relevant to your study. If you are not sure if a list item applies to your research, read the appropriate section before selecting a response.

Materials & experimental systems

n/a	Involved in the study	
☐	☒	Antibodies
☐	☒	Eukaryotic cell lines
☒	☐	Palaeontology and archaeology
☐	☒	Animals and other organisms
☒	☐	Human research participants
☒	☐	Clinical data
☒	☐	Dual use research of concern

Methods

n/a	Involved in the study	
☒	☐	ChIP-seq
☒	☐	Flow cytometry
☒	☐	MRI-based neuroimaging

Antibodies

Antibodies used	All antibodies used, and the suppliers, are described in Supplementary table 1. They were all obtained from Cell Signalling.
Validation	Commercial antibodies were validated by the manufacturer. The Cell Signalling website states "To ensure product performance, we validate all of our antibodies, in-house, in multiple research applications." Antibodies were also validated by the experimenter by using appropriate negative and positive controls (e.g. specific inhibitors/activators of the target protein).

Eukaryotic cell lines

Policy information about cell lines

Cell line source(s)	INS-1 832/13 cells were originally developed by Prof Claes Wollheim (Geneva) and they were supplied by Patrik Rorsman (Oxford). They can be be obtained commercially from Sigma-Aldrich (product code SCC208).
Authentication	INS-1 832/13 cells were authenticated functionally by examining glucose-stimulated insulin secretion, and genetically by examining expression of beta-cells genes such as Pdx1, Nkx6.1, MafA, MafB and Pax6.

Mycoplasma contamination	Cell lines were tested for mycoplasma when they first arrived in the laboratory and were found to be negative.
Commonly misidentified lines (See ICLAC register)	No commonly misidentified cell line was used in the study

Animals and other organisms

Policy information about studies involving animals; ARRIVE guidelines recommended for reporting animal research

Laboratory animals	We used male and female mice, on the C57BL6/J background strain, which are wild-type or carry the Kir6.2-V59M and RIP2-CreER transgenes, as indicated in the Methods. We crossed mice carrying the RIP2-Cre ER transgene with mice carrying the floxed stop Kir6.2 V59M transgene to obtain mice heterozygous for both the RIP2-Cre ER transgene and floxed stop Kir6.2 V59M transgene. After tamoxifen activation at 12 weeks of age, the Kir6.2-V59M transgene is expressed specifically in the pancreatic beta-cells, resulting in rapid diabetes. Mice were used 2 weeks after gene induction, when free-fed blood glucose levels were above 25mM. Mice were maintained on a 12hr light-dark cycle at 21°C and 45-55% relative humidity. They had unrestricted access to water and a regular chow diet (63% carbohydrate, 23% protein, 4% fat; Special Diet Services, RM3).<br>All animal studies were approved by the Department of Physiology Anatomy and Genetics (University of Oxford) ethical review committee and were conducted in accordance with the UK Animals (Scientific Procedures) Act (1986).
Wild animals	No wild animals were used in this study
Field-collected samples	No field collected samples were used in this study
Ethics oversight	All animal procedures were approved by the Department of Physiology, Anatomy and Genetics local ethical review committee. They were conducted in accordance with the UK Animals (Scientific Procedures) Act (1986) under a project licence granted by the UK Home Office.

Note that full information on the approval of the study protocol must also be provided in the manuscript.
